# Miscellaneous Notices

**Published:** 1843-09

**Authors:** 


### Miscellaneous Notices.

### AMERICAN SOCIETY OF DENTAL SURGEONS.

*Fourth Annual Meeting.*—This association commenced its sittings in the city of Baltimore, on the 18th of July, 1843, at 10 o'clock, A. M. in the Assembly Rooms, corner of Holliday and Fayette streets.

Members present during the session—H. H. Hayden, M. D., D. D. S., President; Lewis Roper, M. D., one of the Vice-Pres't; Solyman Brown, M. D., D. D. S., Recording Secretary; Chapin A. Harris, M. D., D. D. S. Corresponding Secretary; Leonard Mackall, M. D., Treasurer; Jahial Parmly, D. D. S. of New York; David Randolph Parmly, New York; Edward Maynard, M.D., D.D.S. Washington City; Enoch Noyes, M.D. Baltimore; T. B. Hamlin, Wytheville, Va.; Charles Rowall, New York; Alexander Neilson, Albany, N. Y.; Edward Laroque, Baltimore; Wm. H. Dwinelle, Cazenovia, N. Y.; Jas. Alcock, D. D. S. New York; H. N. Fenn, M.D. Rochester, N. Y.; J. W. Howlet, D. D. S. Greensboro', N. C. J. S. Gunnel, M. D. Washington City; Sam'l W. Stockton, Philadelphia; Amos Westcott, M.D., D.D.S. Syracuse, N.Y.; Geo. Merryman, Baltimore; Oliver Holmes, Baltimore.

After reading and approving the minutes of the last meeting, Dr. C. A. Harris delivered the opening address, which was ordered to be printed in the Journal with the thanks of the Society.

This was the first occasion since the formation of the Society, on which ladies, including the wives and daughters of some of the members, graced and animated the meeting by their presence.

*Tuesday afternoon, 4 o'clock.*—On motion resolved that Drs. E. Parmly, S. Brown and E. Baker, be continued a committee to prepare a memorial to be presented to the Legislature of the State of New York, asking for a charter of this Society, and to hold property of the value of fifty thousand dollars, for the purpose of erecting a building in the city of New York, as the depository of its library, and other property.

On motion of Dr. Holmes, resolved that the thanks of this Society be presented to Drs. Taylor and Allen, of Cincinnati, for their kind invitation to hold the next meeting of the society in that city, informing them that the society has deemed it expedient for the present to meet in the city of New York.

On motion of Dr. Westcott, resolved that a committee of three be appointed to report at the next meeting on the subject of the Ligamentum Dentis.

E. Maynard, E. Parmly and A. Neilson were that committee.

*Wednesday Morning.*—Dr. Bond one of the professors of the Baltimore

College of Dental Surgery, read a Dissertation on the Morbid Sympathies between the mouth and other parts of the body, which was ordered to be printed with the thanks of the society.

On motion of Dr. Harris, resolved that this society regards the use of mineral paste in plugging carious teeth as malpractice, and that a committee of three be appointed to receive information and facts on that subject, to be transmitted to Dr. Westcott, of Syracuse, in the state of New York, to be by him laid before the Medical Society of the county of Onandaga in that state, before which the subject aforesaid is now pending.

S. Brown, E. Parmly and J. Foster of New York, are that committee.

The members added to the society at this annual meeting, are as follows.

L. S. Allen, Winchester, Va.; Charles F. Martin, Norfolk, Va.; Charles Walker, M. D., Northampton, Mass.; John M. Howe, New York; Wm. Brown Gildea, St. Louis, Mo.; Edward Hale, St. Louis, Mo.; Elijah Bryan, New York; Samuel S. Hornor, Philadelphia; Dr. Ballard, Tunis, Africa; B. R. Robinson, D. D. S. Baltimore; J. A. Pleasants, Richmond, Va.; John B. Rich; New York; A. Robinson, Wheeling, Va.; ——— Porter, Bridgeport, Conn.; R. Woofendale, New York; Ed. Laroque, Baltimore; Geo. Merryman, Baltimore; A. W. Briscoe, Baltimore; J. W. Howlet, D. D. S., Greensborough, N. C.; Charles Linthicum, D. D. S., Baltimore.

*Thursday Morning* 9 *A. M.*—Dr. Westcott read a Dissertation on carious teeth, and the action of sundry agents upon these organs as determined by a a series of experiments by him conducted, during the last year.

The thanks of the society were presented to Dr. Westcott for this Essay, and the same was ordered to be printed.

On motion resolved that a committee of three be appointed with instructions to provide and recommend a plan for guarding the society from imposition in the admission of members.

Drs. Westcott, Noyes and Mackall are that committee.

On motion resolved that the Treasurer be instructed to make out the bills for annual dues, and all other indebtedness of the members to this society, and present them immediately for liquidation.

The officers of the last year were all re-elected.

The Editors of the Journal for the coming year, are S. Brown, C. A. Harris and E. Maynard.

S. Brown was appointed to give the opening address at the next meeting of the society, and the following gentlemen were appointed to read dissertations.

Drs. Maynard, Laroque, Dwinelle, Westcott and J. D. McCabe.

*Thursday Evening.*—The society met at the residence of Dr. Hayden in N. Charles street.

Dr. Gunnel read a paper on the cutting of the Dentes Sapientiæ, which was ordered to be printed with the thanks of the society.

The society adjourned to meet at the American Hotel in the city of New York, on the third Tuesday in July next, 1844, at 10 o'clock, A. M.

SOLYMAN BROWN, *Rec. Sec'ry.*

THE LAST IMPORTATION OF TRANSATLANTIC QUACKERY, RESHIPPED FOR ENGLAND, WITH THE USUAL ALLOWANCE OF "DRAW-BACK."

The *famous,* or more properly, the *infamous* Monsieur Mallan, of mineral paste notoriety, the worthy successor of the Messieurs Crawcour, has recently taken French leave of his tailor, his shoemaker, his upholsterer, his printers, *"et id omne genus,"* who have allowed him to run up bills without footing them, during the past two years in the city of New York.

The profession throughout the country are indebted to some of their enterprising brethren of that city, for having made such an *expose* of the malpractices of this consummate quack, as to induce the good people of New York to be on their guard in relation to employing a foreign mountebank, instead of the honorable members of the dental profession, who are located among them.

It will be remembered by most of our subscribers, that a circular was forwarded to them about two years ago, containing extracts from English journals, together with affidavits taken in New York, with respect to the practices and professions of the individual named above. Such was the effect of this circular as to prevent the empirical vagabond from gaining a footing in any other place than New York, where he was reduced to the alternative of either flight or starvation.

Such is the salutary effect of associated effort in suppressing imposition in dental practice. We hope our brethren in all parts of the country, will exhibit equal enterprise, in every similar case, to the end that the empiricals of the old world may learn to expect no success in the new. The editors of some of the London Journals deserve our thanks for warning the American public of the approach of the above named charlatan to our shores; and we hereby reciprocate the favor by informing them that he has lately returned to their island.

*Dr. B. Randolph Robinson,* graduate of the Baltimore College of Dental Surgery, intends taking passage on board the barque Louisa, which is to sail in a few days for Valparaiso, South America, which place he proposes to make the future field of his professional labor. Few men have enjoyed more ample opportunities for thoroughly qualifying themselves for the profession of dental surgery than he has done, and few, if any, have profited more by them. And, uniting as Dr. R., does, to eminent professional abilities, the courteous, honorable and high-minded gentleman, he could hardly fail any where to win the confidence and patronage of an enlightened and discriminating public. In leaving the circle of his friends and the scenes of his home and youth, to take up his abode among strangers and in a far distant country, he carries with him our best wishes for his future happiness and success.—*Balt. Ed.*

*Incorruptible Teeth.*—We have recently received from Dr. J. Alcock, of New York, several sets of incisor and cuspidati teeth, which for beauty and resemblance to the natural organs, are unsurpassed by any which we have ever seeen. Dr. A. certainly ranks among the very first manufacturers of incorruptible teeth in the world.

*British Dental Journal.*—If any of our subscribers desire to take the above named periodical, the New York editor will supply them at the book-store prices, or four dollars per volume. The numbers of the current volume appear in London, in March, June, September and December. The arrival of the second number is expected daily. OTIS CLAPP, of Boston, is the American publisher.

*Artificial Teeth.*—The first complete double set of artificial teeth worn in the United States, is said to have belonged to Aaron Burr; and to have been manufactured in Paris. We suppose they were constructed similarly to those represented in a plate in Fouchard's "Chirurgien Dentiste," inasmuch as little or no improvement was made in this department of the art, from the time of this celebrated practitioner up to nearly the close of the last century. But subsequently, and particularly during the last twenty years, the achievements in mechanical dentistry, or that branch of the art to which the prosthesis of the teeth belongs, have been most astonishingly great.

*Notice.*—The newly elected members of the American Society of Dental Surgeons, are informed that they can receive their Diplomas by applying to either of the Secretaries. The cost of the Diploma is ten dollars, and the annual dues five dollars payable in advance.

*To Correspondents.*—The letter of Dr. G. G. Brewster, of Portsmouth, N. H. to the senior editor, was not received until after the time when the writer intended it to be used. Dr. B. will see from the account of transactions at the late Annual Meeting of the American Society of Dental Surgeons, that a committee was appointed to take into consideration and report upon the subject in which he feels so warm and commendable an interest. The valuable suggestions of Dr. G. will not, we trust, be lost upon the committee.

M.

The letter from Dr. J. C. M. will appear in the next number.

*Dr. Westcott's Essay on Caries of the Teeth.*—The highly intelligent and enterprising author of this treatise, has opened a new and beautiful field of inquiry, in relation to the decomposition and destruction of the teeth of man, for which our age and country are so unfortunately remarkable.

While some laborious and learned writers are controverting the mooted question, whether caries of teeth is internal or external in its inception—hereditary or superinduced—the effect of external agencies, or systematic disease, Dr. Westcott proceeds directly to the experimental investigation of the chemical agencies of all those substances which are liable to be brought into contact with the human teeth during the ordinary life of man. Having instituted numerous carefully conducted experiments, and opened the way for increasing the number during the coming year, he is preparing himself to present definite results, which will be of the greatest value to those individuals who are anxious at all periods of life, to use proper measures for the preservation of the organs of mastication. And there can be no doubt that there are many such persons in all the upper classes of society in every civilized nation of Europe and America.

The most important instruction which the community at large will derive from the course of experiments referred to, relates to the occasions on which the welfare of the teeth depends on a careful cleansing of the mouth after certain kinds of medicine and diet. If, for example, it be found that the acid of the dried grape or raisin, acts with peculiarly destructive energy on the calcarious enamel and bony structure of the teeth, the lesson inculcated by Dr. Westcott's experiments, will be, that every person who eats this fruit, should invariably rinse and wash the mouth and teeth immediately afterwards, in order to remove this acid; for many substances which would destroy the teeth, might not injure in the least the coats of the stomach or other tissues of the animal economy.

There can be little question of the fact, that many persons injure their teeth incurably, without even so much as suspecting the mode in which they do it. The object of the dental journal, the dental society, and every honorable dental practitioner, should be to enlighten public opinion on this subject. But public opinion can be reached only through the dental profession in this respect. Dentists themselves must first understand the evil and the remedy, before they can instruct their patients and the public generally.

In the present condition of the profession, it may safely be averred, that not one dentist in three can allege any satisfactory reason why the teeth should be cleaned at all, excepting that they may present a more beautiful appearance, or that tartar may not undermine and destroy the gums. The action of various kinds of medicines, drinks and diets, is almost wholly unknown, and thence they are utterly unable to respond to the often repeated question—"How can it be that my teeth have decayed so rapidly?" The dentist is often assured by his patients that their parents had such fine teeth that they took them all with them to their graves; and they wonder how it has happened that, before thirty years of age, all their own teeth have crumbled and disappeared.

Dr. Westcott's experiments will enable himself and his readers to reply to some of these inquiries in a manner which will be wholly satisfactory to every reasonable mind. They will show with the conclusiveness of demonstration, that the teeth are soluble in many of the acrid fluids which are introduced into the mouth, in connexion with the daily habits of the individual; and they will show, furthermore, what are the substances in which these fluids are contained.

If we are permitted to make any suggestions on this subject, in regard to the future experiments of the able individual who has taken this subject in hand with such zeal and perseverance; we would solicit that he so classify his trials as to be able to say positively whether the action of the various solvents of the teeth be in inverse proportion to the age of the subject from whose mouth the teeth are taken, or whether the solvency depends on some other law. In other language, are the teeth of children, as a general rule, more readily destroyed by corrosive agents than those of youths; and are the teeth of persons in middle life more liable to be affected by foreign agencies than those of later years. These are questions, the proper solution of which will be regarded as of incalculable value to all mankind.

That differences in the susceptibility of the teeth of different individuals to the action of solvent agents, are owing to the differences that exist in their density, as is stated by Dr. Harris in his treatise on dental surgery, there can be no doubt, but it would be interesting to the profession to know how much this is influenced by age. That the organs for a time, gradually acquire an increase of density after their eruption, there can be no question, but to what extent, and how long they continue to increase in hardness, yet remains to be shown, as well as the difference between the density of the temporary and permanent teeth. On all of these subjects, the experiments of Dr. W. will doubtless throw much valuable light.

We conclude by expressing the hope that Dr. Westcott will go on with his experiments with renewed zeal after the very kind reception which his essay experienced in the society to which it was addressed. Within a few hours after it was read, the Faculty of the Baltimore College of Dental Surgery conferred upon him unanimously the degree of Doctor of Dental Surgery.

B.

---

*Dr. Bond's Essay on the Sympathy subsisting between the Teeth and other parts of the human system.*—The attention of the profession is respectfully called to this excellent production, as well in regard to the essential importance of the subject to the dental practitioner, as to the lucid and truly scientific manner in which it has been treated by its talented author. Dr. Bond who is one of the professors in the Baltimore Dental College, the son of the venerable Thomas E. Bond, M. D., of the city of New York, was well known to be fully qualified for the task, when he was invited by a resolution of the society, at its annual meeting in Boston, to give an essay on the subject of Dental Sympathy at the next session.

Inasmuch as the society deemed this subject of sufficient importance to warrant its reference to a specially qualified physiologist, and as he has performed his task with great credit to himself, and to the entire gratification of the society which requested it, we trust the truths which it inculcates will commend themselves to all our professional brethren, in such a manner as to be practically useful. B.

---

*Correspondence.*—The propriety of publishing the following letter to the Society, separately from the minutes of the last meeting, to which it might seem appropriately to belong, will be acknowledged, we hope, when we suggest that we have a desire to offer a few editorial remarks on one of the topics to which it briefly alludes.

MY DEAR SIR: *Painesville, July 11th,* 1843.*

From the time that I took leave of you last year, in Boston, there has been no period or event to which I have looked forward with so much interest and pleasure, as that of meeting you again this season, in the city of Baltimore, surrounded by the members and friends of our institution, whose professional and personal characters I so highly appreciate and value; but an illness, as disastrous as severe, occurred in my family, which completely broke up all my plans for the summer, and made it necessary that I should remove from the city as soon as possible, it not being safe, under existing circumstances, any longer to remain there, and travelling was recommended as the most efficient means to restore that health that had been so unexpectedly invaded. On our way we were delayed several days on account of illness, in which I have been a partaker, and we did not, until yesterday, reach this place, which is the end of our journey.

I pray you to present my most friendly regards to all the gentlemen that may be assembled on the present occasion, and express to them the deep regret which I feel in not being able to meet and unite with them in advancing the interests of a cause so dear to our thoughts, so honorable to our pride, so creditable to our ambition, and so well deserving our highest efforts to foster and build up, not only for the good of suffering humanity, and for the profession in which we are engaged, but also to do away with that prejudice in the minds of our fellow citizens, that has hitherto, with its dark and withering influence, thrown a shade of distrust over all the individual efforts that have been made to protect our rights, or guard the public from imposition and abuse.

I do not know of any one in this country, whose individual efforts did more to make the public respect our profession or value our services, than the labors of the late Dr. Hudson, of Philadelphia—and as he is now gone from us, I have thought that the dentists throughout the United States, out of respect for his character and regard for his high professional merit and distinction, would feel happy in having an opportunity of contributing something towards erecting a stone to his memory, with a suitable inscription, which would, while they testify to the high character which he attained by his talents, faithfulness, and industry, also show forth their high appreciation of the character and the value they set upon his example of truth and honesty while engaged in his professional labor.

I think, if a notice was given in the journal, and a treasurer appointed, a sufficient amount would be collected by our next meeting to show the respect of our professional brethren for the character of their late distinguished fellow laborer in the field of dental science. If such a thing should be thought advisable, I would suggest that a neatly printed certificate should be offered to each one, showing that he was a contributor to the erection of a stone to the memory of Hudson.

That the same peace, good will, and unity which have distinguished all our meetings may prevail, is the sincere and ardent wish of

Your greatly obliged friend, E. PARMLY.

To H. H. HAYDEN, M. D.

*President of the Am. Soc. of Dental Surgeons.*

The erection of a suitable monument to the memory of the late Dr. Hudson, of Philadelphia, is a tribute so justly due to his exalted merits as a dental operator, that its propriety cannot be questioned for a moment; but in regard to the *mode* of doing it there may be room for discussion. That the members of the profession, generally, will gladly and liberally contribute to such an object, there is not a question, provided the position in which the memorial should be placed could in any degree comport with the character of him whom it should commemorate, as well in regard to its permanency, as its scenic accompaniments and accessories. In case the remains of the lamented Hudson shall have been deposited where such a superstructure would be either improper or impossible, a site for the proposed monument might be obtained in the new and beautiful rural cemetery attached to either of our large Atlantic cities.

But, it has been suggested that the memory of Dr. Hudson might be more effectually perpetuated by the publication of his likeness in the Journal, as recommended by the Society at its second annual meeting. A committee was then appointed, with instructions to obtain, if possible, the loan of a portrait of this distinguished dentist, for that purpose. This tribute of respect to his memory might, and we doubt not would, be more gratifying to the profession than the erection of a marble or granite monument, which the larger number of our brethren would never have an opportunity of seeing.

We do not intend in this place to attempt his eulogy. We shall leave that task for wiser heads and better hearts. But we confidently reiterate the opinion that some tribute of respect not merely *ought* to be, but *will* be, offered by the American Society of Dental Surgeons, to the memory of the departed Hudson. B.

